# Scléromalacie post traumatique

**DOI:** 10.11604/pamj.2017.28.116.12267

**Published:** 2017-10-06

**Authors:** Mohamed Elyadari, Amina Berraho

**Affiliations:** 1 Service d’Ophtalmologie B, Hôpital des Spécialités, CHU Ibn Sina Rabat, Quartier Souissi, 6220 Rabat, Maroc

**Keywords:** Scléromalacie, traumatisme, mélanome, Scleromalacia, trauma, melanoma

## Abstract

We here report the case of a 18-year old patient who was referred for evaluation of pigmented subconjunctival tumor of the left eye mimicking uveal melanoma. The patient reported that the lesion had evolved rapidly during the previous months. Eye exam showed prominent dark brown lesion adjacent to the limbus between 3 and 9 hours, measuring 10 mm x 6 mm (A). Ultrasonographic biomicroscopy revealed cystic structure communicating with the vitreous cavity, suggesting the diagnosis of uveal hernia rather of melanoma. During a more thorough interview, the patient reported that he had undergone transfixing keratoplasty for cloudy patch secondary to blunt trauma from stone’s throw dating back 10 years. We assumed that the scleral wall had been damaged, leading to the development of scleromalacia with uveal hernia. The patient had recently had an acute episode of vomiting which could have lead to an increase in lesion size. The patient was followed up for several weeks, during which the lesion was stable. The patient underwent surgery with successful uveal tissue reintegration and sclera closure (B).

## Image en médecine

Il s’agit d’un jeune de 18 ans qui a été adressé en consultation pour une tumeur sous conjonctivale pigmentée de l'œil gauche mimant un mélanome uvéal. Le patient a indiqué que la lésion a évolué rapidement au cours des mois précédents. L'examen oculaire a révélé une lésion saillante brun foncé adjacente au limbe entre 3 et 9 heures, mesurant 10 mm sur 6 mm (A). La biomicroscopie ultrasonographique a révélé une structure kystique avec communication avec la cavité vitréenne, faisant penser au diagnostic d’une hernie uvéale plutôt qu’un mélanome. Lors d'un interrogatoire plus minutieux, le patient a déclaré avoir bénéficié d’une kératoplastie transfixiante pour une taie cornéenne séquellaire survenant à la suite d’un traumatisme contusif par jet de pierre datant de 10 ans. Nous avons retenu l'hypothèse que la paroi sclérale avait été endommagée, ce qui avait conduit au développement d’une scléromalacie avec hernie du tissu uvéale. Le patient avait récemment eu un épisode grave de vomissements, ce qui aurait pu entraîner une augmentation de la taille cette lésion. Un suivi a été assuré pendant plusieurs semaines, période au cours de laquelle la lésion était stable. Le patient a bénéficié d’une chirurgie qui a permis la réintégration du tissu uvéal et la fermeture de la sclère (B).

**Figure 1:**
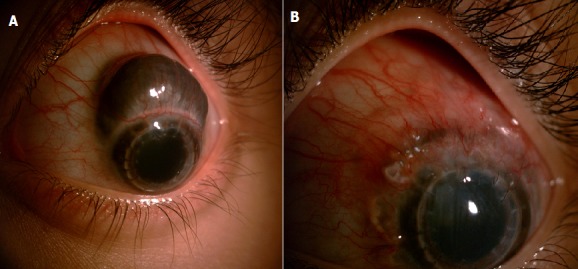
A) scléromalacie avec hernie du tissu uvéale; B) aspect postopératoire

